# Shared Decision Making Assistant (SDMA) and other digital tools for choosing antipsychotics in schizophrenia treatment

**DOI:** 10.1007/s00406-023-01712-9

**Published:** 2023-11-29

**Authors:** Stefan Leucht, Spyridon Siafis, Alessandro Rodolico, Natalie L. Peter, Katharina Müller, Jakob Waibel, Wolfgang Strube, Alkomiet Hasan, Ingrid Bauer, Peter Brieger, John M. Davis, Johannes Hamann

**Affiliations:** 1 Present Address: Department of Psychiatry and Psychotherapy, School of Medicine and Health, Klinikum rechts der Isar, Technical University of Munich, Ismaningerstr. 22, 81675 Munich, Germany; 2 German Center for Mental Health (Deutsches Zentrum für Psychische Gesundheit, DZPG), Munich, Germany; 3 Psychiatry and Psychotherapy, kbo-Isar-Amper-Klinikum München, Munich, Germany; 4 Department of Psychiatry, University of Chicago at Illinois, Chicago, USA; 5 Psychiatry, Bezirkskrankenhaus Mainkofen, Deggendorf, Germany; 6 Psychiatry, Psychotherapy and Psychosomatics, University Augsburg, Augsburg, Germany

It is estimated that the time from research evidence to implementation in clinical practice is on average about 17 years [1]. The reasons for this are complex. But one reason is certainly that evidence is not made available to physician colleagues and patients in a form that they can easily interpret. Clinicians are extremely busy and usually do not have the time to read scientific journals. But based on already outdated estimates, a general practitioner would need to read 19 articles a day, 365 days a year, to cover the relevant new evidence [2]. Even if they did read so many publications, the articles would be difficult to understand because of the jargon used in scientific journals. Not to mention patients, although, according today’s guidelines, they should be involved in decisions in the form of shared decision making. Guidelines are very useful, but their recommendations can often only provide a certain corridor. In areas where there is a wide range of available medications with varied efficacy and side-effect profiles such as antipsychotics for schizophrenia, it is particularly difficult to choose the most suitable medication for a patient. It is almost impossible for the clinician to know all the specifics of each antipsychotic. Moreover, considering the individual preferences of patients within this vast amount of information is a formidable challenge. In order to fill this gap, digital presentation is the way to go.

**Fig. 1:**
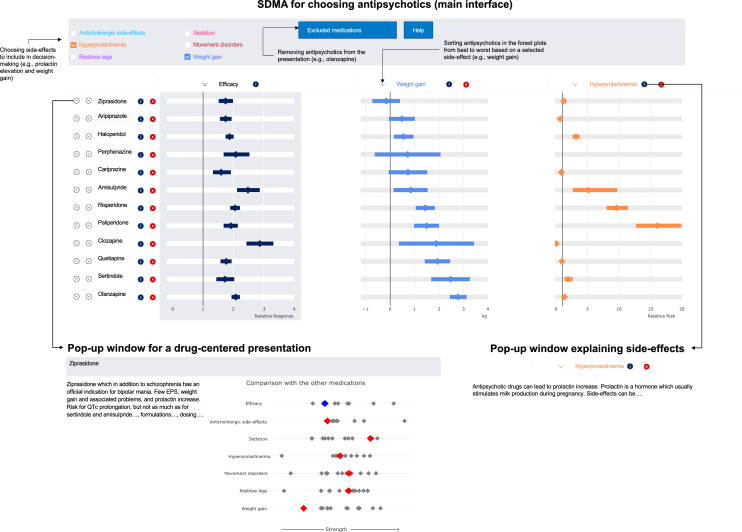
The interfaces of Shared Decision Making Assistant

One such visualization is the Shared Decision-Making Assistant (SDMA). High quality evidence from a network meta-analysis on the efficacy and side-effects of antipsychotics [3] is visualized in a simple form, i.e., interactive forest plots. Physician and patient can first take out the medications that are certainly out of the question, e.g., olanzapine in a patient with diabetes. Then they can pick which of the side-effects among extrapyramidal symptoms, akathisia, sedation, weight gain, prolactin increase, anticholinergic side-effects to include in the decision making alongside overall efficacy. For example, they can consider weight gain and prolactin elevation to be important for the selection of the antipsychotic. With one click, they can then sort the results compared to placebo by outcome. If the priority is for instance on preventing weight gain, then they can sort the results with one click for which drug has the least weight gain compared to placebo at the top. The hierarchies for other outcomes, i.e., efficacy and prolactin elevation in this case, are automatically rearranged and they can see where a drug stands in this comparison (see Fig. 1).

With one click, patients can also find out what the side effects mean. For example, there are no good randomized data on the relationship between antipsychotics and their risk for certain sexual side effects. Therefore, prolactin elevation had to be used as an outcome, and what prolactin is all about is explained. Other elements include a presentation centered on one drug, how it compares to others in terms of efficacy and side effects. And for each drug, a brief text "in a nutshell" is displayed on the essential information such as efficacy, side effects, dose, metabolism, interactions. This multi-layered and easily accessible information saves the medical user from having to look up the product information. We will regularly update our network meta-analysis so that users have up-to-date data and information on new drugs coming to the market.

Pillinger and others [4] have presented a similar tool, which provides data on more side effects and older drugs. We have refrained from doing so because in our opinion the evidence on other side effects and other drugs than those included in the SDMA is much weaker. Unfortunately, this is not always accurately expressed in the strength of evidence according to the GRADE approach used in Huhn et al. [3]. Furthermore, the tool by Pillinger et al. [4] like older visualisations by van Dijk et al. [5] and by Henshall et al. [6] allows patients to specify their individual weighting for different side effects. An algorithm weighs the risk for the various side-effects and the importance a given patient assigns to them and displays the best choice. This approach was also piloted in the development of SDMA, but ultimately discarded. Because such weighting presupposes that, for example, twice as many patients with sedation is the same as twice as many patients with significant weight gain. Moreover, the aim of SDMA is not to propose on single “best option” to patients and physicians (“computerized paternalism”) but rather to stimulate evidence-based discussions within a shared decision-making process.

SDMA is the first such tool the utility of which is being evaluated in a randomized trial [7]. A preliminary version is freely available on our homepage https://ebmpp.org/tools/sdma-app. It is currently available in English, German, Italian, Greek, Japanese and Chinese.

Finally, with this framework, we would like to mention a product under development, PROTECTS_SE, by Rodolico et al. (personal communication), which provides patients and physicians with accurate, evidence and guideline-passed recommendations for managing side effects when they occur.

Although the use of digital tools in the selection of antipsychotics are an obvious support for evidence-based treatment, they are still surprisingly underdeveloped (8). We hope that the aforementioned tools will be further developed and improved in the future to support patients and physicians in shared decision making.
